# Beyond malignancy: the role of carbohydrate antigen 125 in heart failure

**DOI:** 10.1186/2050-7771-1-25

**Published:** 2013-08-30

**Authors:** Chung-Lieh Hung, Ta-Chuan Hung, Yau-Hui Lai, Chi-Sheng Lu, Yih-Jer Wu, Hung-I Yeh

**Affiliations:** 1 Departments of Internal Medicine and Medical Research, Mackay Memorial Hospital, Taipei, Taiwan; 2 The Institute of Health Policy and Management, College of Public Health, National Taiwan University, Taipei, Taiwan; 3 Department of Medicine, Mackay Medical College, New Taipei City, Taiwan; 4 Department of Health Industry Management, Kainan University, Taoyuan, Taiwan; 5 Mackay Junior College of Medicine, Nursing and Management, New Taipei City, Taiwan

## Abstract

Carbohydrate antigen 125 (CA-125), traditionally a tumor marker for screening, diagnosis, and monitoring in ovarian malignancy, had recently been shown increasing evidence and more extensively recognized/explored as a novel surrogate of heart failure (HF). The exact mechanisms underlying the pathophysiologic link between elevated serum CA-125 concentration and HF may be multi-factorial, with both mechanical and inflammatory process including numerous potential cytokines involved. Accumulating data had consistently indicated its diagnostic and prognostic role in HF patients in various clinical settings, however, there is limited clinical information regarding the incremental value or head-to-head comparison of such marker to other well-established HF markers. In this brief review, we aimed to discuss the biosynthesis, and potential insights of underlying pathophysiologies associated with CA-125 secretion in the scenarios of cardiac structural/functional alterations and HF, and further explored its current usage and roles in several recent reports.

## Brief introduction

**Figure 1:**
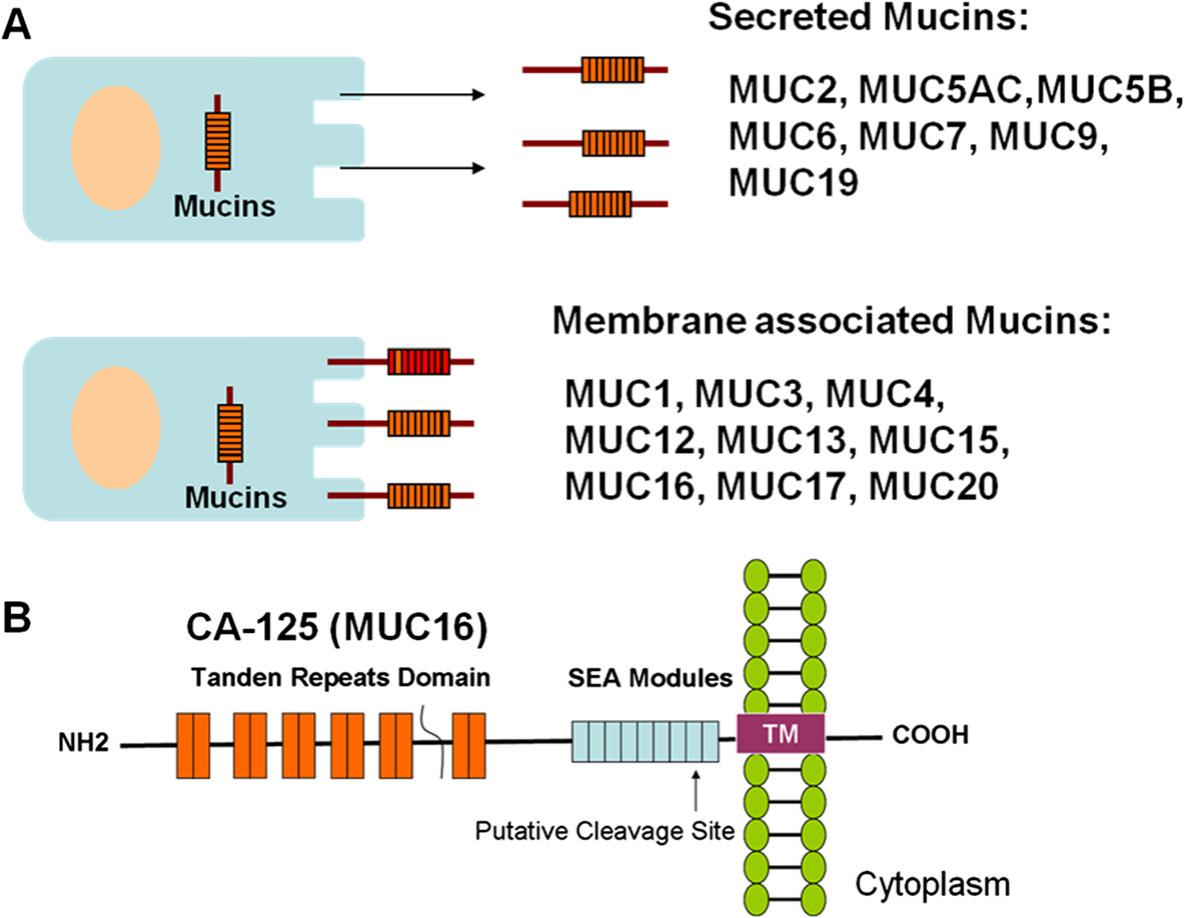
**The Mucin family glycoproteins. (A)** There are two classes of mucins generated in the cells: secreted mucins and membrane associated mucins. **(B)** Diagram of the structure of CA-125. CA-125 is encoded by the *MUC16* gene which is located on human chromosome 19. CA-125 contains a tandem repeat domain that has repeating sequences high in serine, threonine and proline and is highly O-glycosylated. The C-terminal domain of CA-125 contains multiple extracellular SEA (**s**ea urchin sperm protein, **e**nterokinase, and **a**grin) modules, a transmembrane (TM) domain, and a cytoplasmic tail. CA-125 is also thought to be putatively cleaved at a site in the SEA modules.

Carbohydrate antigen 125 (CA-125), also known as MUC16, is a glycoprotein belonging to the mucin (MUC) family [1]. Humans CA-125, encoded by MUC16 gene, contains about 22,000 amino acids and is heavily glycosylated at the extracellular region (Figure 1), which can be released from the cell surface by undergoing proteolytic cleavage and hence released into body fluid, including blood, pleural effusion, and ascites. CA-125 was first detected in ovarian cancer cell line. Subsequent studies showed that it is normally expressed on the surface of cells derived from coelomic epithelium, including pleura, epicardium, fallopian tubes, endometrium, and endocervix. With rich oligosaccharide chains, the physiological role of CA-125 is considered to protect the epithelial luminal surfaces from physical stress through hydration or lubrication process [2,3].

### The role of CA-125 in malignancy and non-cardiac pathophysiology

Clinically CA-125 has long been used as a marker for ovarian cancer [4-6]. In patients with ovarian cancer, accumulated evidence showed that serum levels of CA-125 is helpful in the monitoring of the cancer and further aid in risk stratification and prognosis after treatment [7,8]. However, a rise of serum CA-125 level is not limited to ovarian cancer alone. Other gynaecological malignancies or non-gynaecological malignancies including lung cancer, mediastinal teratoma, and even non-Hodgkin’s lymphoma, have been reported to be associated with a high serum CA-125 level [9-11]. Apart from malignancies, elevation of CA-125 can been seen in situations involving several physiologic or pathologic conditions including early pregnancy, menstruation, peritoneal trauma, and ascites of any cause [12,13]. Hepatic cirrhosis as a cause of CA-125 elevation, for example, had been mentioned to be the most frequent diagnosis in male patients^7^. Another example is that elevation of CA-125 may happen in subjects with pleural effusion [14]. Based on these findings, clinical interpretation of elevated CA-125 should be exercised with caution, in part may be due to its restriction in specificity of diagnosing a single clinical disease entity or scenario.

### The link between CA-125, left-sided cardiac structural/functional alterations and heart failure

Since CA-125 is expressed in epicardium, it is not surprising that cardiac disorders may be associated with elevated CA-125 levels in the blood. Nagele and associates firstly reported the findings of clinical or hemodynamical relevance of several tumor markers, with a special focus on CA-125, in patients with chronic heart failure before and after heart transplantation [15]. Duman et al. ever described a weak association between CA-125 level and right side pulmonary artery pressure, though in that case no association was found between CA-125 level and left-side cardiac hemodynamic (mostly diastolic) parameters [16]. Similar finding was reported by Kouris et al. [17]. What may merit noteworthy is that, Duman et al. also showed that CA-125 was associated with left atrial volume as a supplement to the severity indices of diastolic dysfunction along with BNP levels [16]. In our recent work, we demonstrated that in subjects with HF but preserved LVEF (HFpEF), CA-125 was actually associated with LA remodeling in terms of higher volume rather than other parameters, which may highlight that clinical significance or findings that diastolic impairment or left sided hemodynamic disturbances linked to CA-125 may actually come from left atrial remodeling [18]. On the contrary, D'Aloia et al. demonstrated an association between CA-125 level and both right heart hemodynamics and left ventricular diastolic function [19]. Compared to the former study, data reported by D'Aloia et al. had larger total patient population across a wide range of HF severity in terms of NYHA functional class (Fc) (Duman et al.: 49, Kouris et al.: 77 versus D'Aloia et al.: 286) leading to larger amount of Fc IV population (Duman et al.: 16, Kouris et al.: 16 versus D'Aloia et al.: 83) in D'Aloia's series, and potentially higher number of subjects (28/49 versus 152/286) having CA-125 above normal range (35 U/ml). Differences in total patient numbers, disease severity and distributions may in part explain the potential differences in such correlations. In the same study, D'Aloia also reported that subsequently lower CA-125 at follow up was related to clinical improvement by NYHA class as a positive response to therapy. Again, another study by Vizzardi et al. with larger patient number enrolled and wider range of clinical HF subjects (n = 200, NYHA Fc II-IV) reported that both systolic and diastolic indices, cardiac diameter, and diastolic functional parameters correlated with CA125 level [20]. In one study, CA-125 distinguished subjects with acute decompensated HF [21], and further showed prognostic significance (Table 1). The predictive value for all-cause mortality up to 6-months after index HF hospitalization had also been demonstrated by the same study group [22]. On the other hand, there also seems to be a moderate correlation between CA-125 and brain natriuretic peptide (BNP) level, the most commonly used biomarker reflecting degree of excessive ventricular wall stress, hemodynamics or ventricular filling abnormalities in left-sided HF [23,24]. Interestingly, though both markers had been reported to rise in acute heart failure, combination of them in clinical use may further improve risk stratification at follow up [23,25]. Nevertheless, the elevation of CA-125 in the context of left-sided HF had been proposed to be associated with disease severity in terms of NYHA functional class, [20,22] echocardiographic parameters, and clinical conditions such as effusions or edema [13,23].

**Table 1:** Comparisons of previous CA-125 related heart failure research and reports from previous studies

	**Subjects number**	**HF presentations**	**Clinical status**	**Left side hemodynamics**	**Outcomes available**
Kouris et al. [17]	N = 77	AHF	V	V	?
Nunez et al. [21]	N = 1,111	AHF	V	?	V
Nägele et al. [15]	N = 71	CHF	V	?	V
D’Aloia et al. [19]	N = 286	CHF	V	—	V
Vizzardi et al. [20]	N = 200	CHF	V	V	?
Duman et al. [16]	N = 49	CHF	V	V	?
Chen [24]	N = 285	CHF	V	V	?
Yilmaz et al. [28]	N = 150	All comers	?	V	V
Hung et al. [18]	N = 35	HFpEF	X	V	V

*V*, Positive Finding, *—*, Negative Finding, *?*, Not Reported.

### The clinical associations between CA-125 and right-sided heart failure

Significant elevation of CA125 level in a patient having atrial septal defect (ASD) with right ventricular dilatation was ever reported by Mathew et al. [26]. The association between CA125 and right ventricular parameters, either structural or functional, in subjects presented as chronic obstructive pulmonary disease (COPD) had also been described [27,28]. In Yilmaz’s work, 40 patients with another age- and gender-matched control group with detailed transthoracic echocardiography were examined [28]. They observed that patients with higher systolic pulmonary artery pressure as well as lower tricuspid annular plane systolic excursion or worse tricuspid annulus systolic velocity (S’) all had significantly higher CA-125 levels than did those without. In another retrospective work (totally 150 patients enrolled) by Yilmaz et all reported that elevated CA-125 level was associated with worse left ventricular ejection fraction and positively correlated with systolic pulmonary artery pressure [29]. In particular, patients with right ventricular dilatation had significantly higher CA-125 levels compared to those without. They also concluded that patients with high CA-125 level encountered more frequent hospitalization, atrial fibrillation and mortality at future follow up. This work actually pointed out the potential link between bi-ventricular failure and elevated CA-125 level was not confined to only right or left ventricle alone.

### Possible mechanisms linking elevated CA-125 to clinical heart failure

**Figure 2:**
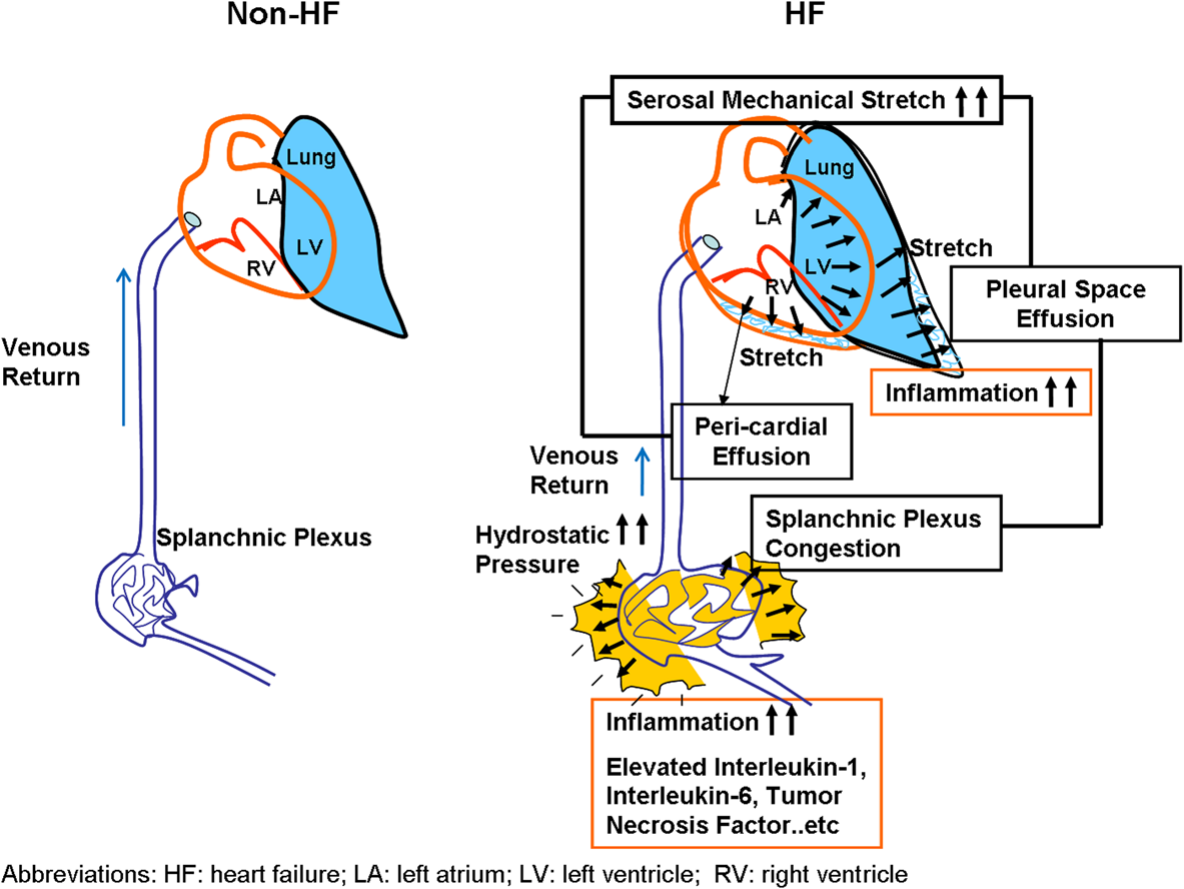
**Illustrations regarding how HF may cause elevated hydrostatic pressure and congestion, leading to both serosal mechanical stretch and third space fluid retention with resultant inflammation and cytokines release.** These complex chain reactions were believed to be the main cause of systemic CA-125 release.

Interpretation of elevated CA-125 levels in HF should consider the triggering factors as well as the production sites. These issues have been investigated and no simple answer was found. So far, production of CA-125 in subjects with HF had been hypothesized to happen with so-called “stressed” mesothelial cells, in response to both hemodynamic and inflammation stimuli [30]. It had been proposed that fluid overload accompanied by high venous pressure due to heart failure may increase the congestion and hydrostatic pressure in mesothelium, [31] which may actually provoke the release of several inflammatory markers such as interleukin-6 (IL-6), interleukin-10 (IL-10), and tumor necrosis factors. In this regard, serosal irritation due to inflammation process, mechanical stress, or fluid congestion all might stimulate mesothelial cells to initiate CA-125 synthesis [32,33]. The synthetic capacity of mesothelium of CA-125 had been demonstrated by Zeillemaker et al., who investigated the secretion pattern of CA-125 using mesothelial cell monolayer as an in vitro model [34] by utilizing several inflammatory cytokines including interleukin-1 (IL-1), tumor necrosis factor-α and lipopolysaccharides as stimuli. Apical secretion of CA-125 was shown in 6 hours, with the most effective stimuli observed to be IL-1. Congestion as a hallmark of clinical HF manifestation may involve pulmonary congestion/edema, pleural effusion and ascites, which are believed to be interrelated with systemic inflammation activation as a vicious cycle [35]. The formation of congestion itself as a consequence of HF had made the splanchnic bed with an abundance of mesothelial cells exposed to high-venous pressure and ascitis (Figure 2). Furthermore, the combination of both pathological process may thus further contribute to neurohumoral-axis activation facilitating CA-125 production [36]. Additionally, the possible translocation of bacteria or endotoxin formation during the exacerbation of HF, a condition most likely to happen in subjects presenting with right heart failure as well as gastrointestinal functional impairment in the context of bowel congestion with HF, may also play a key role in the “hyper-response” of CA-125 from mesothelium [37].

On the other hand, the hypothetical link between pericardial stimuli and elevation of CA-125 in subjects with HF had also been observed. Seo et al. [38] ever reported a 59% positive staining in the pericardial mesothelial cells, while Soma et al. reported a negative finding [39]. More or less, accumulating evidence had indicated the mesothelium (pericardial, pleura or peritoneum) as a potential primary source of CA-125 in HF or other non-malignant clinical scenarios [40,41], while data regarding the severity or extent of underlying stimuli, inducing media or modalities, and whether such elevation in HF was also secondarily influenced by effusions *per se* or other associated cytokines still remained a debate [41]. Perhaps, all these possibilities exist, but just contribute differentially according to the severity and etiologies of HF. To clarify these issues, experiments studying animal models of HF mimicking different presentations of human HF encountered clinically may provide the solution. This approach will determine the major production sites of CA-125 as well as the major stimuli in various stages of HF.

### Potential clinical application of CA-125 in HF

The range of value and potential clinical implication of CA-125 regarding its predictive value in monitoring HF beyond and the correlates with other well-established HF markers (e.g. BNP or NT-ProBNP) remained largely unknown. In general, serum CA-125 level seemed to be 7-fold higher (HF: 105.2 ± 139, range 4.6 to 1169.5 U/ml vs Control: 14.9 ± 22 U/ml, respectively) in subjects with acute HF, 2.5 times in chronic HF (HF: 68 ± 83, range 3 to 537 U/ml) [23] and nearly 1.5 times higher in female HFpEF (HFpEF: 17.6 ± 10.2, range 4.94 to 104.3 U/ml) [18], the value actually had wide range across a heterogeneous spectrum in specific subgroups of HF patients [12,26,29,32,39]. Previous work on healthy volunteers showed that serum CA-125 level differences may exist between healthy pre- (mean level: 12.6 U/ml) and post-menopausal women (10.5 U/ml) though without statistical significance, with significant lower value was observed in healthy men when compared to pre-menopausal women (9.2 vs 12.6 U/ml, p = 0.007) [42]. Serum CA-125 had shown significant correlation with BNP level, elevated LV end-diastolic filling pressure and larger LA volume in systolic HF [16], so far there is a paucity of data on the incremental value of CA 125 when added on BNP. Though comparable long-term (14 ± 2 months) predictive value for CA 125 when compared to NT-ProBNP in stable, chronic systolic HF had recently been tested in a feasibility study with much lower cost (about 10 times less expensive for CA 125 vs NT-ProBNP, roughly U.S. $1.07 vs $10.74) [43]. The lack of change in serum CA 125 concentration rather than NT-ProBNP seemed to confer higher risk of death in systolic HF during a 3-month therapeutic interval. [44] In addition to the potential clinical application in patients with systolic heart failure, CA125 may help to clarify the uncertain management of patients with HFpEF [45]. The incremental value of CA 125 superimposed on NT-ProBNP in female HFpEF was proved in our work recently [18]. Interestingly, all these studies showed statistically significant association between NT-ProBNP and CA-125 level. However, all these reports were of smaller sample size and used a diversity of clinical outcomes as end-points. Thereafter, a large scale study with specific HF patients population and well-defined clinical outcomes may be necessary for a head-to-head comparison between CA-125 and other commonly used HF biomarkers.

## Conclusion

With better understanding of the underlying biological mechanisms and pathophysiological processes of heart failure in the recent era, biomarker as a surrogate in disease screening, diagnosis, or as a guide to effects of therapy or outcomes had broadened the utilization of these tools by physicians in clinical settings. The incremental clinical value by using multi-biomarker panel looking into potentially diverse pathways or biological signaling involved in a clinical disease entity are evolving. CA-125, the glycoprotein primarily used for ovarian cancer screening or therapeutic monitoring, is gaining more and more attention as another role in heart failure. Insights into the understanding of its role and mechanisms involved in heart failure had made it different from other biomarkers for the same purpose, though questions and issues regarding the real determinants for such glycoprotein release, short- and long-term monitoring for treatment, and the potential costs as a routine marker for heart failure may worth further investigation.

## Competing interests

The authors declare that they have no competing interests.

## Authors’ contributions

CLH drafted the manuscript. TCH and YHL had made substantial contributions to conceptual framework of this review. CSL had participated the graphing of CA125 molecular structure and figure edition. YJW involved in the drafting of the manuscript. HIY have given final approval of the version to be published. All authors read and approved the final manuscript.
